# A Novel One-Dimensional Chaotic System for Image Encryption Through the Three-Strand Structure of DNA

**DOI:** 10.3390/e27080776

**Published:** 2025-07-23

**Authors:** Yingjie Su, Han Xia, Ziyu Chen, Han Chen, Linqing Huang

**Affiliations:** 1 School of Advanced Manufacturing, Guangdong University of Technology, Jieyang 522000, China; 3122008941@mail2.gdut.edu.cn (Y.S.); 3122008947@mail2.gdut.edu.cn (H.X.); 3223008668@mail2.gdut.edu.cn (Z.C.); 2 School of Marine Science and Technology, Shanwei Institute of Technology, Shanwei 516600, China; hanchengdut@gmail.com

**Keywords:** one-dimensional chaotic system, SHA-256 hash function, three-stranded structure of DNA, security analysis

## Abstract

Digital images have been widely applied in fields such as mobile devices, the Internet of Things, and medical imaging. Although significant progress has been made in image encryption technology, it still faces many challenges, such as attackers using powerful computing resources and advanced algorithms to crack encryption systems. To address these challenges, this paper proposes a novel image encryption algorithm based on one-dimensional sawtooth wave chaotic system (1D-SAW) and the three-strand structure of DNA. Firstly, a new 1D-SAW chaotic system was designed. By introducing nonlinear terms and periodic disturbances, this system is capable of generating chaotic sequences with high randomness and initial value sensitivity. Secondly, a new diffusion rule based on the three-strand structure of DNA is proposed. Compared with the traditional DNA encoding and XOR operation, this rule further enhances the complexity and anti-attack ability of the encryption process. Finally, the security and randomness of the 1D-SAW and image encryption algorithms were verified through various tests. Results show that this method exhibits better performance in resisting statistical attacks and differential attacks.

## 1. Introduction

With the rapid development of information technology, digital images are increasingly widely used in various fields, such as mobile devices, the Internet of Things, medical imaging, and other fields. The transmission and storage volume of image data have grown explosively. However, image data usually contains a large amount of sensitive information, such as personal privacy, business secrets, military strategies, etc. Therefore, protecting the security of image data has become a crucial issue.

Image encryption technology is one of the key means to protect the security of image information. Through encryption, the original image can be converted into a ciphertext image that cannot be directly recognized. Only users with the correct key can decrypt and restore the original image. This encryption mechanism can effectively prevent unauthorized access, tampering and leakage, ensuring the security of image data during transmission and storage. Although image encryption technology has made remarkable progress, it still faces many challenges.

On the one hand, attackers can utilize more powerful computing resources and more advanced algorithms to crack encryption systems, making traditional encryption algorithms vulnerable when facing attack methods such as brute-force cracking, differential attacks, and known plaintext attacks. Some simple encryption methods can be easily cracked by attackers through statistical analysis or pattern recognition, thereby restoring part or all of the information of the original image. On the other hand, image data usually has the characteristics of high resolution and large capacity, which requires encryption algorithms to have efficient encryption and decryption speeds while ensuring security. However, many highly secure encryption algorithms often have high computational complexity, resulting in a long encryption and decryption process and making it difficult to meet the real-time requirements.

To address the above challenges, it is particularly important to develop an image encryption algorithm that is efficient, secure and has strong anti-attack capabilities. In recent years, chaos theory and DNA coding technology have received extensive attention in the field of image encryption. Chaotic systems have characteristics such as initial value sensitivity, randomness and unpredictability, and can provide good randomness and complexity for image encryption. DNA coding technology utilizes the structure and characteristics of DNA molecules to encode image data into DNA sequences, thereby achieving encryption. By combining chaotic systems with DNA coding technology, the advantages of both can be fully exerted to design a new type of image encryption algorithm to meet the high security requirements in the current field of image encryption.

The main contribution of this paper are as follows.

1. A new one-dimensional chaotic system (1D-SAW) based on the sawtooth wave function and the Wang–Chen algorithm was designed. By introducing nonlinear terms and periodic disturbances, this system is capable of generating chaotic sequences with high randomness and initial value sensitivity.
2. A new diffusion rule based on the three-strand structure of DNA is proposed. Compared with the traditional DNA encoding and XOR operation, this new diffusion rule can further enhance the complexity and anti-attack ability of the encryption process.
3. Complete tests were conducted on the 1D-SAW and image encryption algorithm from multiple perspectives, verifying the security and randomness of them.

The structure of this article is organized as follows: Section 2 provides an overview of the relevant work in the field. Section 3 introduces existing DNA coding techniques and the triplex structure of DNA. Section 4 details the proposed new chaotic system. Section 5 presents the mapping rules based on the triplex structure of DNA. Section 6 describes the image encryption method based on the proposed 1D-SAW system. Section 7 experimentally verifies the effectiveness of the proposed method. Section 8 provides a comparative analysis of the proposed encryption algorithm with other recent algorithms. Finally, Section 9 concludes the paper by summarizing the contributions and findings.

## 2. Related Works

In 1949, Shannon proposed that the design framework of cryptography should be divided into two fundamental components: confusion and diffusion, thereby laying a solid foundation for the theoretical development of cryptography [1]. In 1998, J. Fridrich pioneered the application of chaotic systems to digital image encryption by constructing a scrambling-diffusion framework for image encryption, in which the values generated by chaotic systems were utilized to drive the scrambling process within the encryption algorithm [2].

Since then, digital image encryption systems based on chaos have predominantly adopted the scrambling–diffusion structural design originally proposed by Shannon [3,4,5,6,7]. Liu et al. proposed an encryption scheme that combines a new 3D inverse proportional chaotic map (3D-IPCM) and a pseudorandom number generator (PRNG) that passes rigorous tests. This scheme employs a chromosome crossover-based scrambling algorithm and an efficient diffusion algorithm, achieving strong security and low computational cost, making it suitable for IoT scenarios [3]. Erkan et al. conducted an image encryption scheme termed OSMRD-IE, which employs a 2D hybrid Michalewicz–Ackley (2D-HMA) map to perform octal-based shuffling and multilayer rotational diffusion [5]. This approach enhances security by mitigating the limitations of existing methods through dynamic chaotic perturbations and robust parameter control mechanisms. Le et al. introduced a simultaneous permutation and diffusion framework (SPDF) for medical image encryption based on a new 1D chaotic map (1D-CICMIC), which enhances security through dynamic scrambling and embedded diffusion, and demonstrates high efficiency and robustness against common attacks [7].

Combined with chaotic systems, DNA coding, as an effective coding method, has been widely used in the design of image encryption algorithms [8,9,10,11,12,13,14,15]. Gao et al. introduces a novel 3D memristive cubic map with dual discrete memristors (3D-MCM) that exhibits richer dynamical behaviors and higher complexity compared to single-memristor systems and demonstrates its application in image encryption through the quaternary-based permutation and dynamic emanating diffusion (QPDED-IE) scheme, showing strong resistance to cryptanalytic attacks [8]. Deb et al. proposed a new image encryption method, which combines the cyclic block function with the DNA-based linear feedback shift register (LFSR) to improve security and efficiency [10]. Zhao et al. proposed a flexible multi-image encryption scheme using a new n-dimensional chaotic model and eight-base DNA operations, capable of encrypting any type, quantity, and size of images, with enhanced security and anti-attack capabilities through unique key generation and SCAN-based scrambling [13]. Du et al. presented a hybrid one-dimensional and two-dimensional cross-feedback hyperchaotic system framework combined with diffusive DNA coding operations for digital image encryption, enhancing key security and the statistical relationship between plaintext and ciphertext [14]. In 2025, a novel parallel color image encryption algorithm based on the 2D logistic-Rulkov neuron map (2D-LRNM) is proposed, which enhances security through cross-channel information interaction and block-wise parallel encryption, while significantly reducing computation time by leveraging parallel computing resources and overcoming the limitations of traditional Logistic maps [15].

## 3. Preliminaries

### 3.1. DNA Encoding

Deoxyribonucleic acid (DNA) is a double-stranded helical structure composed of four bases: adenine (A), thymine (T), cytosine (C), and guanine (G). According to Ref. [16], these four bases pair through hydrogen bonds. A pairs with T, and C pairs with G, forming base pairs. In cryptography, binary data is usually mapped to DNA base sequences and encoded by taking advantage of the complementary base pairing characteristics of DNA [17]. A DNA coding rule is shown in Table 1.

### 3.2. Triplex DNA Structure

**Figure 1:**
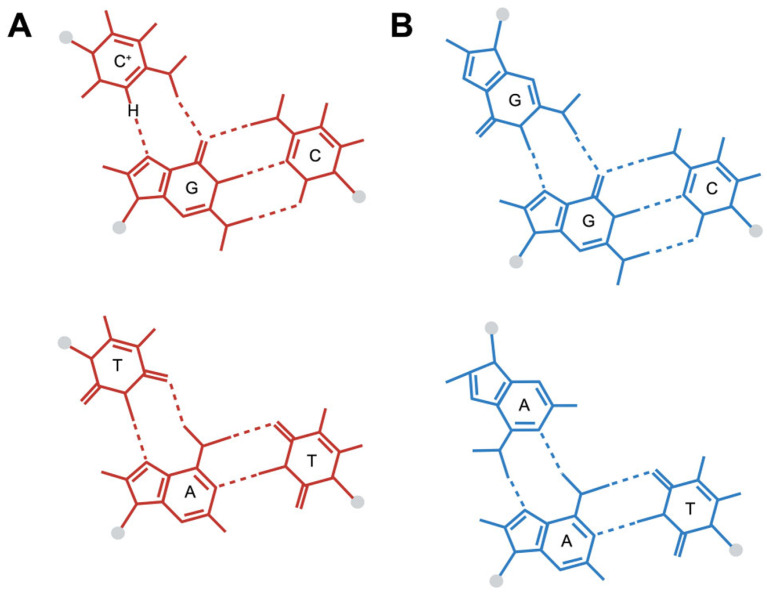
(**A**): A DNA three-stranded structure containing two triplet base pairs, C^+^-G-C and T-A-T, (**B**): A DNA three-stranded structure containing two triplet base pairs, G-G-C and A-A-T. (created in BioRender. Hisey, J. (2024). https://BioRender.com/f14l364, accessed on 26 November 2024).

The triplex DNA structure is a special DNA conformation composed of three DNA strands, which is different from the common double-stranded DNA. The formation of this structure depends on specific base sequences and usually involves complementary pairing between single-stranded DNA rich in purines and double-stranded DNA rich in pyrimidines [18]. For example, a single strand rich in adenine can form a hydrogen bond with thymine in a double-stranded DNA rich in thymine, thereby forming a stable three-stranded structure. There are various types of three-stranded DNA, among which H-DNA is formed by repeating sequences of homologous purine/homologous pyrimidine mirror images. In this structure, half of one strand folds back to pair with the double-stranded DNA, forming a reverse-Hoogsteen hydrogen bond, while the other half remains in a single-stranded state. In addition, there are other types such as H-r DNA and H-y DNA, which are folded back from single strands rich in purine or pyrimidine, respectively, to form hydrogen bonds with the corresponding strands in double-stranded DNA. The formation of these structures not only depends on the base sequence but is also affected by factors such as the superhelical state of DNA, pH value, and ion concentration. Figure 1 shows an example of triplex DNA structure.

## 4. The Proposed New Chaotic System

The sawtooth wave function is a periodic function, and its expression is provided by Equation (1):

(1) $$\begin{array}{c} saw(x)=x-T*floor(x/T) \end{array}$$

where *T* is the period of the sawtooth wave.

The theory of the Wang–Chen algorithm is to implement local perturbation on the system by designing an appropriate controller, so that the system generates chaotic behavior. Inspired by sawtooth wave function and Wang–Chen algorithm, a novel one-dimensional chaotic system is conducted.

First of all, design a nonlinear term $rxe^{x}$, which includes the multipliers nonlinear term and the exponential term. The sawtooth wave function is used to provide periodic disturbances for the system. The proposed 1D-SAW is defined by Equation (2):

(2) $$\begin{array}{c} x_{n+1}=rx_{n}e^{x_{n}}+saw\left(rx_{n}\right) \end{array}$$

where *r* is a control parameter and the period *T* of the sawtooth function is set as 1.

### 4.1. Lyapunov Exponent Test

**Figure 2:**
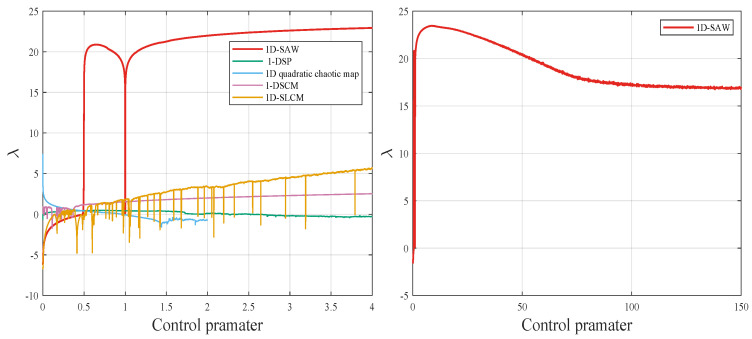
The LE values of 1D-SAW, 1-DSP, 1D quadratic chaotic map, 1-DSCM, and 1D-SLCM.

The Lyapunov exponent (LE) test is a method used to quantify the sensitivity of a dynamical system to initial conditions. It measures the average rate at which nearby trajectories in the system’s phase space diverge over time. A positive Lyapunov exponent indicates that the system exhibits chaotic behavior, while a negative or zero exponent suggests stability or periodicity. The LE value can be calculated by Equation (3):

(3) $$\begin{array}{c} \lambda =\lim_{N\to \infty }\frac{1}{N}\sum_{n=0}^{N-1}\ln\left|\frac{df(x_{n})}{dx}\right| \end{array}$$

where *n* denotes the index of the data points in the generated time series $f(x_{n})$. Recent comparisons of the proposed chaotic system with other chaotic maps including 1-DSP [19], 1D quadratic chaotic map [20], 1-DSCM [21], and 1D-SLCM [22] are illustrated in Figure 2. The 1D-SAW map exhibits a higher LE value over a broader parameter range.

**Figure 3:**
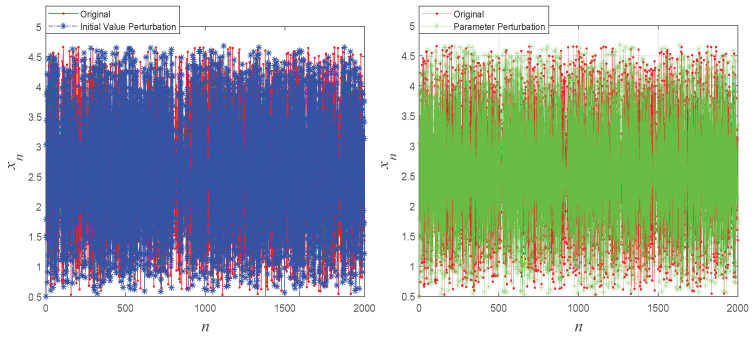
The initial value and parameter perturbation of 1D-SAW.

To visually assess the sensitivity of the proposed system to initial values and control parameters, Figure 3 illustrates the chaotic sequences generated with slight variations in initial conditions and control parameters. This figure highlights the robust sensitivity of the new chaotic maps to both initial values and control parameters.

### 4.2. Bifurcation Analysis

**Figure 4:**
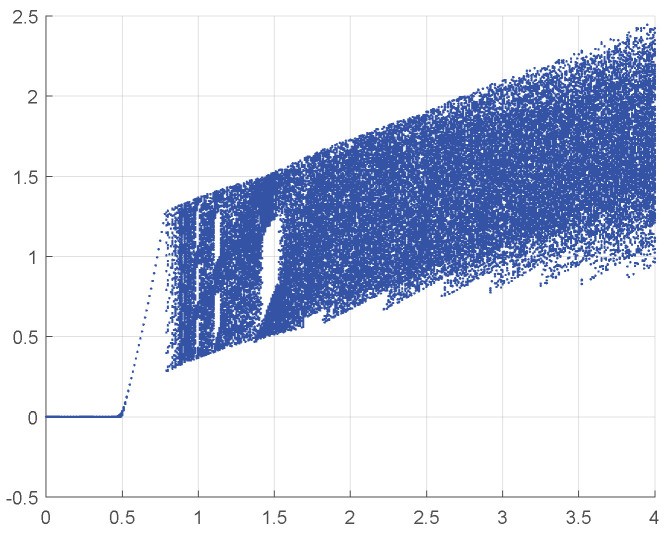
Bifurcation diagram of 1D-SAW.

The bifurcation diagram can reflect the behavior of a dynamical system. From Figure 4, it is evident that 1D-SAW experience a transformation from non-chaotic state to chaotic state when control parameter r∈(0,2). For values r≥2, 1D-SAW consistently exhibits chaotic behavior, indicating that the sequences generated by the 1D-SAW are suitable for cryptographic applications.

### 4.3. Sample Entropy

Sample entropy (*SE*) can be used to measure the self-similarity of the sequences generated by dynamical systems [23]. The calculation method of *SE* is provided by Equations (4)–(7).

(4) $$\begin{array}{c} D_{m}\left(i\right)=\left[X\left(i\right),X\left(i+1\right),\ldots ,X\left(i+m-1\right)\right] \end{array}$$

(5) $$\begin{array}{c} A=\frac{1}{N-m}\sum_{i=1}^{N-m}\frac{1}{N-m-1}A_{i} \end{array}$$

(6) $$\begin{array}{c} B=\frac{1}{N-m}\sum_{i=1}^{N-m}\frac{1}{N-m-1}B_{i} \end{array}$$

(7) $$\begin{array}{c} SE\left(dim,tolerance,N\right)=-\mathrm{lg}\frac{A}{B} \end{array}$$

Here, *A* and *B* are the counts of vectors meet the conditions given in Equation (8).

(8) $$\left\{\begin{array}{c} d[X_{m+1}\left(i\right),X_{m+1}\left(j\right)]<r\\ d[X_{m}\left(i\right),X_{m}\left(j\right)]<r \end{array}\right.$$

**Figure 5:**
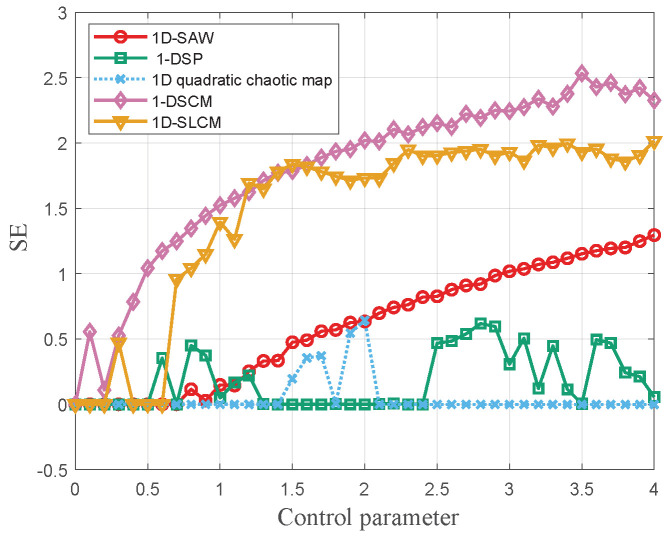
The *SE* values of 1D-SAW, 1-DSP, 1D quadratic chaotic map, 1-DSCM, and 1D-SLCM.

$d[X_{m}\left(i\right),X_{m}\left(j\right)]$ is the Chebyshev distance between X(i) and X(i+m−1). In this experiment the dim and tolerance are set as 2 and 0.2, respectively. From Figure 5, it is observed that 1D-SAW performs better than 1-DSP and 1D quadratic chaotic map.

### 4.4. 0-1 Test

The 0-1 test is a method used to determine whether a time series has chaotic characteristics. It determines the dynamic properties of the system by mapping the time series onto a two-dimensional plane and observing its diffusion behavior on the plane. The value *K* can be calculated by the following equations:

(9) $$\begin{array}{c} p\left(n\right)=\sum_{i=1}^{n}X\left(i\right)\cos ir \end{array}$$

(10) $$\begin{array}{c} s\left(n\right)=\sum_{i=1}^{n}X\left(i\right)\sin ir \end{array}$$

(11) $$\begin{array}{c} M\left(n\right)=\lim_{N\to \infty }\frac{1}{N}\sum_{i=1}^{n}{\left[p\left(i+n\right)-p\left(i\right)\right]}^{2}+{\left[s\left(i+n\right)-s\left(i\right)\right]}^{2} \end{array}$$

(12) $$\begin{array}{c} K=\frac{\log M(n)}{\log n} \end{array}$$

**Figure 6:**
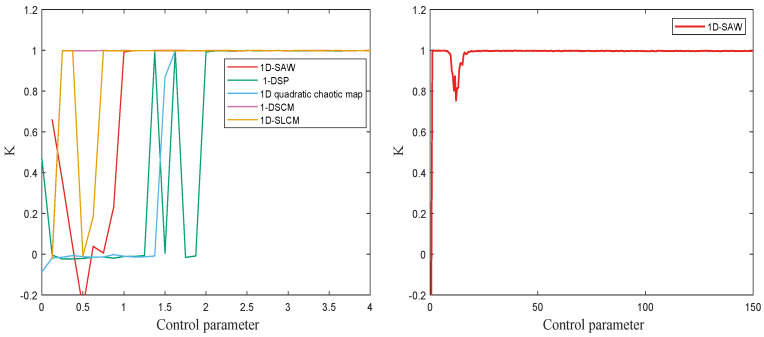
The 0-1 test results of 1D-SAW, 1-DSP, 1D quadratic chaotic map, 1-DSCM and 1D-SLCM.

To avoid bias from a single choice of *c*, the 0-1 test is repeated for 100 random values uniformly distributed in [π/5,4π/5] and report the mean value of *K*. The value *K* is approximated to 1 means the dynamic system exhibit chaotic behavior. As shown in Figure 6, the test statistic *K* remains well below 1 for r∈(0,1.5), whereas it rapidly approaches 1 once r exceeds 1.5, confirming that the 1D-SAW system is chaotic throughout the interval (1.5,15)∪(20,150).

### 4.5. NIST SP 800-22 Test

The NIST (National Institute of Standards and Technology) test is a statistical testing method widely used in randomness detection, mainly for evaluating the randomness of the output sequences of random number generators or pseudo-random number generators. NIST test can determine whether the sequence meets the statistical requirements of randomness. If the sequence passes all the tests, it indicates that its randomness is good, and it is suitable for fields such as cryptography that require high-quality random numbers. In this experiment, we set the significance level α=0.001 and the results is shown in Table 2. The tabulated *p*-values are the minima observed across all sub-tests; since every minimum exceeds 0.01 the overall pass rate is 100%, confirming that the 1D-SAW sequences satisfy the NIST randomness requirements.

### 4.6. Comparative Dynamics Analysis

In this subsection, a comparative analysis of the 1D-SAW system against classical one-dimensional chaotic maps (logistic and tent) is presented in Table 3. The results demonstrate that the 1D-SAW system exhibits a significantly higher maximum Lyapunov exponent, a broader chaotic parameter range, and stronger sensitivity to initial conditions. Moreover, unlike the logistic and tent maps, which exhibit periodic windows and degradation behaviors, the 1D-SAW system remains robustly chaotic across its operational range without notable degeneration.

## 5. DNA Triplex Mapping Rules

The DNA-encoded XOR operation is a simple encryption method that combines DNA encoding and XOR logical operations. First, the pixel values or other digital data of the image are converted into DNA base sequences through specific mapping rules. Next, generate a DNA key sequence of the same length as the image data. Perform XOR operations bit by bit between the encoded DNA sequence and the key sequence. The XOR operation rules are shown in Table 4.

This DNA-encoded XOR operation is characterized by simplicity and efficiency. The XOR operation is fast and suitable for handling encryption tasks of large-scale data. Moreover, due to its reversibility, the same key is used in the encryption and decryption processes, making the entire process very convenient. However, it also has some limitations. Due to the linear nature of the XOR operation, it is vulnerable to linear attacks, and attackers may derive the key based on the known plaintext and ciphertext information.

Inspired by the triplex DNA structure, the mapping rules for DNA triplex diffusion is proposed, which is shown in Table 5. This mapping rule maps each three-base sequence to a specific base, thereby providing a new method for the diffusion operation in image encryption. Compared with the DNA XOR operation, this mapping rule based on the three-stranded DNA structure has some advantages.

In the process of designing the mapping table, the formation and dissociation conditions of the three-stranded DNA structure, such as the hyperhelical state, pH value, ion concentration, etc., were considered, which can increase the complexity of the ciphertext. Furthermore, it also provides a certain theoretical basis for emerging technologies such as future bio-computers.

## 6. Proposed Method

**Figure 7:**
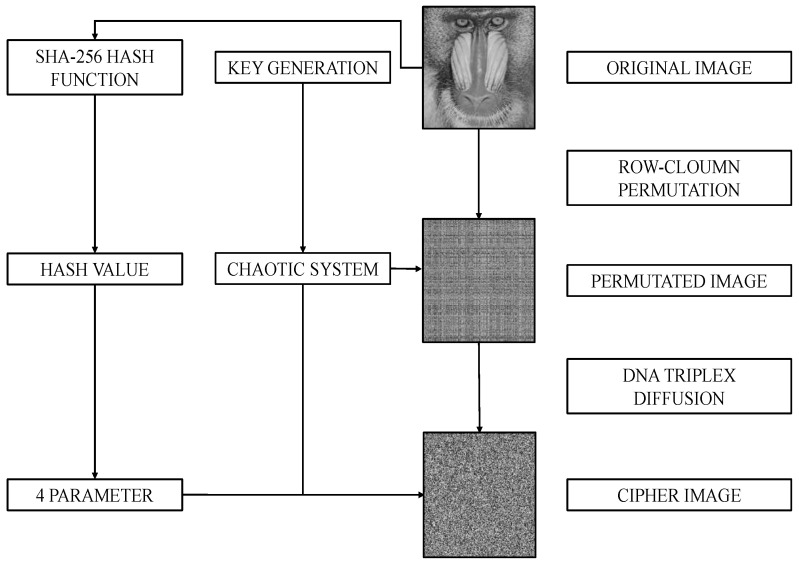
The visual representation of the proposed method.

In this section, an image encryption method based on the 1D-SAW is outlined. The encryption framework consists of three steps: key generation, row–column permutation operation and DNA triplex diffusion operation. The visual representation of the proposed method is provided in Figure 7.

The robustness of the proposed encryption method is predicated upon the secret key, which is constituted by the initial conditions and the control parameter. According to the Lyapunov exponent figure of 1D-SAW (Figure 2), when the control parameter r≈7.9, 1D-SAW exhibits the highest LE value, indicating that 1D-SAW can generate high randomness sequences.

The details of proposed encryption algorithm are as follows.

Step 1: Input the original image into the SHA-256 hash function to obtain the hash value *h*.

Step 2: The hash value *h* will be divided into eight groups (32 bits per group) and get $\left\{h_{1},h_{2},h_{3},h_{4},h_{5},h_{6},h_{7},h_{8}\right\}$.

Step 3: Generate four parameters, which is as delineated by the Equation (13).

(13) $$\left\{\begin{array}{c} p_{1}=h_{1}⊕h_{2}\\ p_{2}=h_{3}⊕h_{4}\\ p_{3}=h_{5}⊕h_{6}\\ p_{4}=h_{7}⊕h_{8} \end{array}\right.$$

Step 4: To enhance the plaintext sensitivity of the algorithm, four initial values $\left\{key_{1},key_{2},key_{3},key_{4}\right\}$ are chosen and influenced by the parameters.

(14) $$\left\{\begin{array}{c} x_{1}=key_{1}\\ x_{2}=key_{2}\\ x_{3}=key_{3}\times p_{1}\times p_{2}\\ x_{4}=key_{4}\times p_{3}\times p_{4} \end{array}\right.$$

Step 5: $\left\{x_{1},x_{2},x_{3},x_{4}\right\}$ are input to 1D-SAW to generate four sequences for $N_{0}$ times. The final value of these sequences are denoted as {x,y,z,w}. Especially, according to Figure 3, $N_{0}$ is set as 2000 to eliminate transient effects.

Step 6: To improve key sensitivity, *z* and *w* will be influenced by *x* and xy.

(15) $$\left\{\begin{array}{c} x=x_{1}\\ y=x_{2}\\ z=x_{3}\times x\times y\\ w=x_{4}\times x\times y \end{array}\right.$$

Step 7: {x,y,z,w} are input into 1D-SAW to generate four random sequences {X,Y,Z,W}. These sequences will be processed using Equation (16).

(16) $$\left\{\begin{array}{c} X=mod(fix\left(X*10^{15}\right),\;M)\\ Y=mod(fix\left(Y*10^{15}\right),\;N)\\ Z=mod(fix\left(Z*p_{1}*p_{2}*10^{15}\right),\;256)\\ W=mod(fix\left(W*p_{3}*p_{4}*10^{15}\right),\;256) \end{array}\right.$$

Step 8: Perform a row–column confusion operation using *X* and *Y*.

Step 9: DNA encode the confused image *P* and chaotic sequences *Z* and *W* using Table 1 and obtain img, $Z_{DNA}$ and $W_{DNA}$, respectively.

Step 10: Perform DNA triplex diffusion operation on img using $Z_{DNA}$ and $W_{DNA}$ and Table 5.

Step 11: Finally, DNA decode the diffused image to obtain the encrypted image *C*.

The sender will send the cipher image *C*, hash value *h*, and Key to the receiver for decryption. The decryption method is the reverse process of the encryption method; the details of encryption and decryption process are illustrated in Algorithms 1 and 2.
**Algorithm 1** Encryption algorithm**Input:** Original image *P* and four random sequences *X*, *Y*, *Z* and *W*.**Output:** 
Encrypted image *C*.  1:**for** i=1:M **do**  2:   t=P(i,:)  3:   P(i,:)=P(X(i),:)  4:   P(X(i),:)=t  5:**end for**  6:**for** j=1:N **do**  7:   t=P(:,j);  8:   P(:,j)=P(:,Y(j))  9:   P(:,Y(j)10:**end for**11:img=DNAencode(P)12:$Z_{DNA}=DNAencode\left(Z\right)$13:$W_{DNA}=DNAencode\left(W\right)$14:**for** i=1:4×M×N **do**15:   Perform DNA triplex diffusion operation on img based on Table 5 and $Z_{DNA},W_{DNA}$16:**end for**17:C=DNAdecode(img)

**Algorithm 2** Decryption algorithm
**Input:** 
The encrypted image *C* and four random sequences *X*, *Y*, *Z* and *W*.**Output:** 
Original image *P*.  1:

img=DNAencode(C)

  2:

$Z_{DNA}=DNAencode\left(Z\right)$

  3:

$W_{DNA}=DNAencode\left(W\right)$

  4:**for** i=1:4×M×N **do**  5:   Perform DNA triplex inverse diffusion operation on img based on Table 5 and $Z_{DNA},W_{DNA}$  6:
**end for**
  7:

P=DNAdecode(img)

  8:**for** i=M:−1:1 **do**  9:   t=P(i,:)10:   P(i,:)=P(X(i),:)11:   P(X(i),:)=t12:
**end for**
13:**for** j=N:−1:1 **do**14:   t=P(:,j);15:   P(:,j)=P(:,Y(j))16:   P(:,Y(j)17:
**end for**



## 7. Results and Discussions

**Figure 8:**
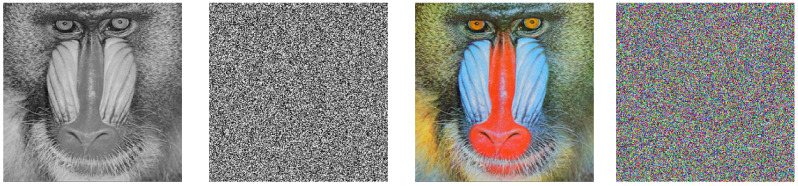
Original images and encrypted images.

The simulations are conducted on the Matlab 2022b software on a personal computer with CPU 2.40 GHz, NVIDIA GeForce RTX 4060 Laptop GPU, 16GB memory, and Windows 11 operating system. The encryption results of the baboon image and cameraman image are depicted in Figure 8.

### 7.1. PSNR Analysis

The peak signal-to-noise ratio (*PSNR*) is widely used for evaluating image quality and quantifying differences between two images. For grayscale images, its *PSNR* value can be calculated using Equation (17).

(17) $$\left\{\begin{array}{c} PSNR=10\mathrm{lg}(\frac{255\times 255}{MSE})\\ MSE=\frac{\sum_{i=1}^{M}\sum_{j=1}^{N}{\left(P\left(i,j\right)-C\left(i,j\right)\right)}^{2}}{M\times N} \end{array}\right.$$

*M* and *N* are the width and height of two images. A reduced *PSNR* value indicates a greater disparity and the *PSNR* values of the proposed method are provided in Table 6 and Table 7. All the *PSNR* values are lower than 10, which suggests that the encryption algorithm provides a higher degree of security.

### 7.2. Differential Attacks

The number of pixel change rate (*NPCR*) and the unified average changing intensity (*UACI*) are usually used to evaluate the sensitivity of encryption algorithms to plaintext changes [24]. The calculation method of *NPCR* and *UACI* are provided by Equation (18).

(18) $$\left\{\begin{array}{c} NPCR=\sum_{i=0}^{H}\sum_{j=0}^{W}D(i,j)\times 100\%\\ UACI=\frac{1}{W\times H}\sum_{i=0}^{H}\sum_{j=0}^{W}\frac{\left|c_{1}\left(i,j\right)-c_{2}\left(i,j\right)\right|}{255}\times 100\% \end{array}\right.$$

Here, *M* and *N* are the width and height of two images. When $c_{1}\left(i,j\right)=c_{2}\left(i,j\right)$, D(i,j)=0 and if $c_{1}\left(i,j\right)\neq c_{2}\left(i,j\right)$, D(i,j)=1. A higher *NPCR* (over 99.6094%) and an appropriate *UACI* value (33.4635%) indicate that the encryption algorithm has good sensitivity to plaintext changes. As shown in Table 8 and Table 9, all the *NPCR* and *UACI* values are closed to ideal value, proving that the proposed encryption model can effectively resist differential attacks.

### 7.3. Exhaustive Attack Analysis

#### 7.3.1. Security Key Space

As highlighted in [25], an encryption algorithm should ideally possess a key space of at least $2^{100}$ The proposed encryption system employs a set of keys defined as $key_{i}\in \left(0,5\right)$, i=1,2,3,4 and $N_{0}\in \left(200,2000\right)$ and r∈(0,150). Assuming a computational precision of $10^{-15}$, the key space can be calculated as follows:

(19) $$keyspace=5^{4}\times 150\times 1800\times {\left(10^{15}\right)}^{4}\approx 2^{227}\gg 2^{100}$$

Given that $2^{227}$ is significantly larger than the recommended threshold of $2^{100}$, this calculation underscores the robustness of the proposed encryption algorithm against brute-force attacks.

#### 7.3.2. Secret Key Sensitivity

In image encryption based on chaotic systems, key sensitivity refers to the extremely high response ability of the encryption algorithm to minor changes in the key. Specifically, even if the key undergoes extremely minor changes, the encrypted image will produce completely different results (Table 10).

### 7.4. Statistical Attack Analysis

#### 7.4.1. Histogram Analysis

**Figure 9:**
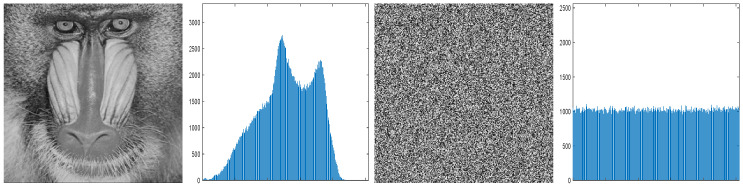
Histogram analysis of baboon.

Histogram testing determines the randomness of encrypted images by analyzing the distribution of gray values. If the encryption algorithm can evenly distribute the gray values of the plaintext image, making the histogram of the encrypted image nearly flat, then it will be difficult for attackers to obtain any useful information about the original image through statistical analysis, thereby enhancing the resistance of the encrypted image to statistical attacks. From Figure 9, the histogram of encrypted image is flat, indicating that it will be difficult for attackers to obtain any useful information about the original image through statistical analysis.

#### 7.4.2. Correlation Coefficient Test

Correlation coefficient test mainly determines the randomness and security of ciphertext images by analyzing the correlation between adjacent pixels, which can be calculated by Equation (20).

(20) $$\left\{\begin{array}{c} r_{xy}=\frac{\mathrm{cov}(x,y)}{\sqrt{D(x)}\times \sqrt{D(y)}}\\ cov\left(x,y\right)=\frac{1}{N}\sum_{i=0}^{N}\left(x_{i}-E\left(x\right)\right)\left(y_{i}-E\left(y\right)\right)\\ D\left(x\right)=\frac{1}{N}\sum_{i=0}^{N}{\left(x_{i}-E\left(x\right)\right)}^{2}\\ E\left(x\right)=\frac{1}{N}\sum_{i=0}^{N}x_{i} \end{array}\right.$$

**Figure 10:**
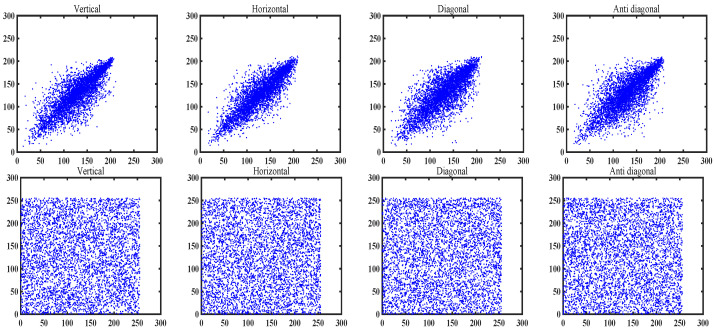
Correlation analysis of original and encrypted baboon image in four directions.

In plaintext images, adjacent pixels usually have a high correlation. However, after the image is encrypted by a secure encryption algorithm, the correlation should be significantly reduced to prevent attackers from using the correlation between pixels to restore image information. In this simulation, 5000 pairs of pixels were chosen from natural image and cipher image. The results of the correlation coefficient are detailed in Table 11 and Figure 10. From Table 11 and Figure 10, the correlation coefficients of all encrypted images are close to 0, indicating that there is no obvious correlation between pixels.

#### 7.4.3. Information Entropy Analysis

Information entropy H(m) is an indicator for measuring the uncertainty or randomness of information and is used to quantify the average amount of information or the degree of uncertainty of a random variable. The calculation of H(m) is provided in Equation (21).

(21) $$H\left(m\right)=\sum_{i=0}^{N}p\left(m_{i}\right)\log\frac{1}{p(m_{i})}$$

$p(m_{i})$ is the probability that the random variable *m* takes the value $m_{i}$, and *N* is the total number of all possible values of *m*. For an 8-bit grayscale image, the grayscale value of each pixel can be an integer ranging from 0 to 255, with a total of 256 possible values. If the gray value distribution of the image is completely uniform, then the information entropy of the image reaches the maximum value of 8. From Table 12 and Table 13, the H(m) is closed to 8, indicating that the gray value distribution of the image is very uniform, and it is difficult to obtain useful information through statistical analysis.

Furthermore, according to Ref. [26], the $\left(k,T_{B}\right)$-local Shannon entropy analyses were carried out for both grayscale and color images. Specifically, we randomly selected *k* non-overlapping image blocks, each with dimensions $T_{B}\times T_{B}$ pixels. For each block, the Shannon entropy was calculated using Equation (21). Finally, the sample mean of the Shannon entropy values across all selected blocks was determined. Here, the *k* and $T_{B}$ is set as 100 and 46, respectively. The results of these analyses are presented in Table 14 and Table 15, which collectively demonstrate the local randomness and information content of the encrypted images. All the values are closed to the ideal value.

### 7.5. Chosen-Plaintext Attack

**Figure 11:**
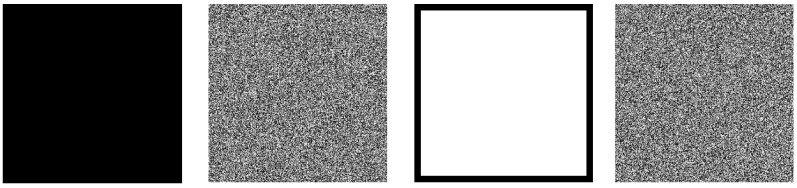
Encryption results for all-black and all-white images.

The chosen-plaintext attack is a type of cryptographic attack method. The attacker can freely choose a specific plaintext and obtain its corresponding ciphertext. By analyzing the relationship between plaintext and ciphertext, attackers attempt to infer the key of the encryption algorithm or its internal structure. In general, a cryptographic system’s ability to produce a noise-like ciphertext image when encrypting an all-black or all-white plaintext image is indicative of its robustness against chosen plaintext attacks (Figure 11 and Table 16).

### 7.6. Cropping and Noise Attack

**Figure 12:**
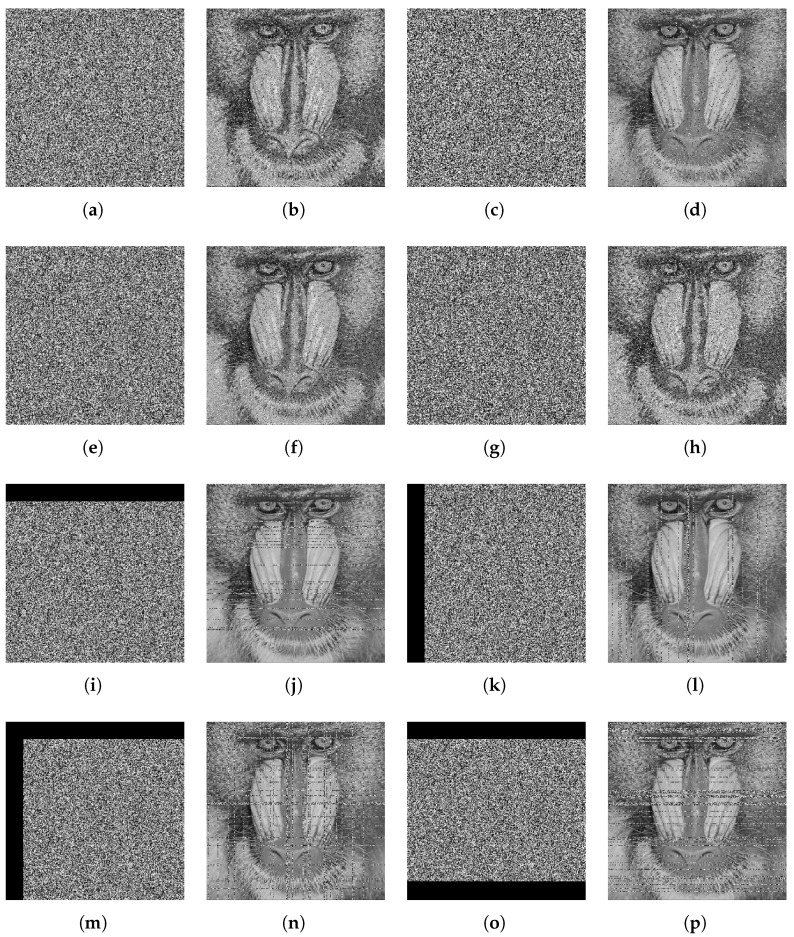
(**a**) 0.01 Gaussian noise attack, (**b**,**d**,**f**,**h**,**j**,**l**,**n**,**p**) the decrypted images, (**c**) 0.01 Salt & Pepper noise attack, (**e**) 0.01 speckle noise attack, (**g**) 0.05 speckle noise attack, (**i**,**k**) 10% unilateral cropping attack. (**m**,**o**) ≈20% multilateral cropping attack.

During the transmission of encrypted data via public channels, the integrity of the data can be compromised due to noise interference or data loss. As illustrated in Figure 12 and Table 17, the proposed algorithm demonstrates significant robustness against such adversarial conditions. Specifically, it maintains the capacity to restore the essential information of the original image, even when the ciphertext image is subjected to noise corruption or partial data cropping.

### 7.7. Time Complexity Analysis

To quantify the computational burden of the proposed encryption algorithm, a detailed complexity analysis in terms of asymptotic time complexity and primitive-operation counts is conducted. For an image of size M×N pixels, the algorithm consists of three sequential phases. The overall encryption complexity is governed by four sequential stages, each linear in image size, yielding an aggregate bound of O(MN). In key-stream generation, the 1D-SAW map is iterated $N_{0}+4MN$ times to eliminate transients and produce four independent sequences X,Y,Z,W; every iteration requires one multiplication, one exponential evaluation, and one sawtooth wave modulo, hence the cost is O(MN). During permutation, rows and columns are swapped exactly once according to *X* and *Y*, amounting to M+N swaps and preserving the O(MN) bound. In the diffusion phase, each pixel is expanded into four DNA bases, producing 4MN table look-ups and bitwise assignments; each of these 4MN DNA symbols is then diffused with two key-stream symbols via the triplex mapping table, an O(1) operation per symbol, so the entire diffusion step remains O(MN). Consequently, the overall encryption time complexity is O(MN).

## 8. Comparative Analysis

To assess the efficacy of the proposed encryption method, a comparative analysis is conducted compared to the recent algorithm. The results are provided in Table 18, including the best performance among the proposed algorithm and other encryption schemes.

From Table 18, the proposed method achieves a *PSNR* value of 7.4795 and an entropy value of 7.9994. Compared with other algorithms, it demonstrates superior performance in resisting statistical attacks.

## 9. Conclusions

This paper introduces an innovative image encryption algorithm that integrates 1D-SAW and DNA triplex diffusion rules, enhancing the complexity and security of the encryption process. Comparative analysis with other recent image encryption algorithms demonstrates that the proposed method achieves superior performance in resisting statistical attacks and ensuring the security of encrypted images. However, while the current algorithm does have significant computational overhead, future research will be committed to addressing this challenge through a combination of algorithm optimization and integration of emerging technologies, such as quantum cryptography and artificial intelligence, to enhance the security and efficiency of image encryption systems.

## Figures and Tables

**Table 1:** DNA encoding rule.

Index	A	T	C	G
Binary value	00	01	10	11

**Table 2:** NIST test results of 1D-SAW.

Test Index	*p*-Value	Result
Frequency	0.779188	PASS
BlockFrequency	0.851383	PASS
CumulativeSums	0.667330	PASS
Runs	0.816537	PASS
LongestRun	0.096578	PASS
Rank	0.115387	PASS
FFT	0.181557	PASS
NonOverlappingTemplate	0.508621	PASS
OverlappingTemplate	0.657933	PASS
Universal	0.048716	PASS
ApproximateEntropy	0.935716	PASS
RandomExcursions	0.384796	PASS
RandomExcursionsVariant	0.383573	PASS
Serial	0.665331	PASS
LinearComplexity	0.419021	PASS

**Table 3:** Comparative dynamics analysis.

Characteristic	1D-SAW	1-DSP	1D Quadratic Chaotic Map	1-DSCM	1D-SLCM
Lyapunov Exponent	High	Low	Low	Medium	Medium
Sample Entropy	Medium	Low	Low	Medium	High
0-1 Test	Pass	Pass	Pass	Pass	Pass
NIST Test	Pass	Pass	Pass	Pass	Pass

**Table 4:** DNA XOR operation.

XOR	A	T	C	G
**A**	A	T	C	G
**T**	T	A	G	C
**C**	C	G	A	T
**G**	G	C	T	A

**Table 5:** Mapping rules for DNA triplex diffusion.

Triplet	Mapped	Triplet	Mapped	Triplet	Mapped	Triplet	Mapped
AAA	A	AAC	C	AAG	G	AAT	T
ACA	C	ACC	A	ACG	T	ACT	G
AGA	G	AGC	T	AGG	A	AGT	C
ATA	T	ATC	G	ATG	C	ATT	A
CAA	C	CAC	A	CAG	T	CAT	G
CCA	A	CCC	C	CCG	G	CCT	T
CGA	T	CGC	G	CGG	C	CGT	A
CTA	G	CTC	T	CTG	A	CTT	C
GAA	G	GAC	T	GAG	A	GAT	C
GCA	T	GCC	G	GCG	C	GCT	A
GGA	A	GGC	C	GGG	G	GGT	T
GTA	C	GTC	A	GTG	T	GTT	G
TAA	T	TAC	G	TAG	C	TAT	A
TCA	G	TCC	T	TCG	A	TCT	C
TGA	C	TGC	A	TGG	T	TGT	G
TTA	A	TTC	C	TTG	G	TTT	T

**Table 6:** *PSNR* values of gray images.

Grayscale Image	Baboon	Airfield	Flower	Fruits	Sailboat	Cameraman
*PSNR*	9.5288	8.4350	8.3866	8.5559	8.2376	7.4795

**Table 7:** *PSNR* values of color images.

Color Image	Baboon	Airplane	Flower	Fruits	Sailboat	Yacht
*PSNR*	8.9433	7.9775	7.9836	7.9016	8.0864	8.4596

**Table 8:** The *NPCR* and *UACI* values for gray images.

Grayscale Image	Baboon	Airfield	Flower	Fruits	Sailboat	Cameraman
*NPCR*	99.6086	99.6071	99.6134	99.6057	99.6133	99.6384
*UACI*	33.4528	33.3936	33.4394	33.4866	33.4155	33.6313

**Table 9:** The *NPCR* and *UACI* values for color images.

Color Image	Baboon	Airplane	Flower	Fruits	Sailboat	Yacht
*NPCR*	99.6363	99.6212	99.6160	99.6246	99.6021	99.5981
*UACI*	33.5126	33.5708	33.4226	33.4791	33.4619	33.4938

**Table 10:** The *NPCR* and *UACI* values for each key with $10^{-15}$ difference or 1 in $N_{0}$.

Image	Key	*NPCR* (%)	*UACI* (%)	Image	Key	*NPCR* (%)	*UACI* (%)
Baboon	$key_{1}$	99.6021	33.4607	Airfield	$key_{1}$	99.6113	33.4288
	$key_{2}$	99.6094	33.4430		$key_{2}$	99.6330	33.5652
	$key_{3}$	99.6204	33.4840		$key_{3}$	99.6166	33.4290
	$key_{4}$	99.6124	33.4370		$key_{4}$	99.5956	33.5812
	$N_{0}$	99.6181	33.4530		$N_{0}$	99.6235	33.5420
Flower	$key_{1}$	99.6179	33.4978	Fruits	$key_{1}$	99.6058	33.4947
	$key_{2}$	99.6210	33.4848		$key_{2}$	99.6060	33.4847
	$key_{3}$	99.6124	33.4621		$key_{3}$	99.6128	33.4885
	$key_{4}$	99.6072	33.4639		$key_{4}$	99.6103	33.4844
	$N_{0}$	99.6083	33.4872		$N_{0}$	99.6099	33.4709
Sailboat	$key_{1}$	99.6150	33.4306	Cameraman	$key_{1}$	99.6216	33.4997
	$key_{2}$	99.6014	33.4240		$key_{2}$	99.5819	33.4379
	$key_{3}$	99.6119	33.4273		$key_{3}$	99.6201	33.5910
	$key_{4}$	99.6147	33.4391		$key_{4}$	99.6155	33.4650
	$N_{0}$	99.5998	33.4655		$N_{0}$	99.5850	33.4757

**Table 11:** The correlation analysis of original images and encrypted images.

Image		Vertical	Horizontal	Diagonal	Anti Diagonal
Baboon	Original	0.7587	0.8605	0.7221	0.7121
	Encrypted	0.0298	0.0095	0.0061	−0.0318
Airfield	Original	0.9439	0.9380	0.9061	0.9076
	Encrypted	0.0197	−0.0199	0.0140	−0.0047
Flower	Original	0.9904	0.9914	0.9820	0.9865
	Encrypted	−0.0104	0.0110	−0.0244	−0.0238
Fruits	Original	0.9841	0.9872	0.9725	0.9792
	Encrypted	−0.0018	−0.0131	−0.0206	0.0067
Sailboat	Original	0.9716	0.9774	0.9553	0.9579
	Encrypted	0.0218	−0.0134	−0.0042	−0.0197
Cameraman	Original	0.9524	0.9155	0.8922	0.9090
	Encrypted	−0.0055	−0.0027	0.0108	−0.0115

**Table 12:** The information entropy value for grayscale images.

Grayscale Image	Baboon	Airfield	Flower	Fruits	Sailboat	Yacht
Original	7.3585	7.1206	7.4107	7.3644	7.4853	7.5603
Encrypted	7.9994	7.9993	7.9993	7.9993	7.9993	7.9992

**Table 13:** The information entropy value for color images.

Color Image	Baboon	Airplane	Flower	Fruits	Sailboat	Yacht
Original	7.7626	6.6639	7.6900	7.5190	7.7632	7.6563
Encrypted	7.9998	7.9998	7.9997	7.9998	7.9998	7.9997

**Table 14:** The local information entropy value for grayscale images.

Grayscale Image	Baboon	Airfield	Flower	Fruits	Sailboat	Yacht
Original	6.6951	5.9774	5.9929	5.5850	6.1039	6.0960
Encrypted	7.9125	7.9104	7.9108	7.9113	7.9113	7.9121

**Table 15:** The local information entropy value for color images.

Color Image	Baboon	Airplane	Flower	Fruits	Sailboat	Yacht
Original	6.6987	5.6542	5.9728	5.4976	6.0775	6.2540
Encrypted	7.9106	7.9123	7.9114	7.9111	7.9109	7.9108

**Table 16:** The *NPCR*, *UACI*, and *PSNR* entropy values for all-black and all-white images.

Image	*NPCR*	*UACI*	*PSNR*	Entropy	Correlation Coefficient		
All-black	99.6201	33.4534	4.7639	7.9993	0.0019 (V)	0.0134 (H)	0.0243 (D)
All-white	99.6120	33.4270	4.7667	7.9994	0.0103 (V)	−0.0029 (H)	0.0086 (D)

**Table 17:** *PSNR* values of cropping and noise attack.

Image	*PSNR*
Figure 12b	15.6709
Figure 12d	19.7472
Figure 12f	17.6552
Figure 12h	15.0703
Figure 12j	19.7227
Figure 12l	19.7073
Figure 12n	16.9327
Figure 12p	16.6524

**Table 18:** Comparative analysis results for the proposed method and other methods.

Algorithm	Plaintext Sensitivity		Key Sensitivity		PSNR	Entropy	Correlation Coefficient			Time
	***NPCR***	***UACI***	***NPCR***	***UACI***			**Vertical**	**Horizontal**	**Diagonal**	
Ref. [21]	100	34.5079	99.6170	33.4580	8.4064	7.9993	0.0010	−0.0053	0.0069	O(MN)
Ref. [22]	99.6098	33.4606	99.6131	33.4771	8.8260	7.9994	−0.0102	−0.0092	0.0034	O(MN)
Ref. [27]	99.7884	33.4768	99.6044	33.4905	8.8260	7.9994	−0.0018	−0.0021	−0.0002	O(MN)
Ref. [28]	99.6038	33.5101	–	–	8.3979	7.9975	0.0031	0.0036	0.0008	O(MN)
Ref. [29]	99.6142	33.4891	99.6093	33.4958	7.8300	7.9992	−0.0014	−0.0027	0.0009	O(MN)
Ref. [30]	99.6158	33.4723	99.6112	33.4769	7.9800	7.9992	−0.0009	−0.0016	0.0005	O(MN)
Ref. [31]	99.6094	33.4635	99.6047	33.4680	8.2100	7.9994	0.0007	−0.0013	0.0004	O(MN)
Ref. [32]	99.6099	33.4656	99.6097	33.2970	9.2272	7.9993	0.0124	−0.0128	−0.0153	O(MN)
Proposed	99.6384	33.6313	99.6330	33.5652	7.4795	7.9994	−0.0018	−0.0131	−0.0206	O(MN)

## Data Availability

The datasets generated and/or analyzed during the current study are available from the https://www.hlevkin.com/hlevkin/06testimages.htm repository (accessed on 18 June 2025).
